# Lipid-Binding Proteins in Brain Health and Disease

**DOI:** 10.3389/fneur.2019.01152

**Published:** 2019-11-07

**Authors:** Miriam Corraliza-Gomez, Diego Sanchez, Maria D. Ganfornina

**Affiliations:** Departamento de Bioquímica y Biología Molecular y Fisiología, Instituto de Biología y Genética Molecular, Universidad de Valladolid-CSIC, Valladolid, Spain

**Keywords:** blood–brain barrier, lipid transport, oxidative stress, neuroinflammation, amyloidogenesis, neurodegeneration, demyelination, lysosomal storage disorder

## Abstract

A proper lipid management is paramount for a healthy brain. Lipid homeostasis alterations are known to be causative or risk factors for many neurodegenerative diseases, or key elements in the recovery from nervous system injuries of different etiology. In addition to lipid biogenesis and catabolism, non-enzymatic lipid-binding proteins play an important role in brain function and maintenance through aging. Among these types of lipoproteins, apolipoprotein E has received much attention due to the relationship of particular alleles of its gene with the risk and progression of Alzheimer's disease. However, other lipid-binding proteins whose role in lipid homeostasis and control are less known need to be brought to the attention of both researchers and clinicians. The aim of this review is to cover the knowledge of lipid-managing proteins in the brain, with particular attention to new candidates to be relevant for brain function and health.

**Graphical Abstract:**
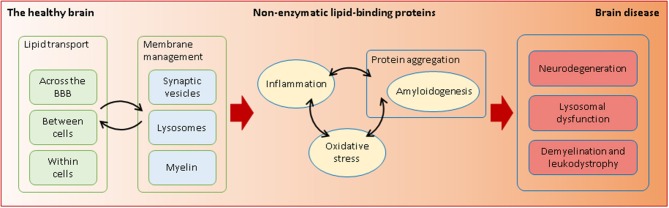
Non-enzymatic lipid-interacting proteins are required in health and disease. Through their transporting and membrane-managing functions, they intervene in processes that are essential for the fragile equilibrium between a healthy and a diseased brain.

## Introduction

Brain is a lipid-enriched organ. Lipids are on high demand in the brain, since they are required for the long expansions of neurons and, massively, for myelin construction. These structures, characteristic of the nervous system, were initially thought to be static assemblies once development was concluded. Now, neuronal processes (dendrites and axons), their synaptic terminals, and their myelin sheaths are known to be constantly remodeling. Their reorganization is the basis of experience-dependent plasticity and, importantly, of the unexpected endogenous abilities of our nervous system to recover from damages, disease, and aging-related deterioration.

It is estimated that around 5% of the genome codes for proteins dealing with lipids, which are estimated to include 10,000 different species (1–3). Anabolic and catabolic routes generate a plethora of individual lipids with energetic, signaling, or structural functions. To accomplish the lipid-related demands of our nervous system, with its particularly relevant membranous structures, we need not only the set of enzymes that generate, transform, or degrade them. Lipids have properties requiring additional managing within a hydrophilic environment. We devote this review to the cohort of proteins in charge of binding, transporting, or presenting lipids to enzymes, defining this functional group under the umbrella of “non-enzymatic lipid managers.”

Taking cholesterol as an example, we can appreciate how lipid metabolism in the central nervous system (CNS) is unique. The brain is the most cholesterol-rich organ in the body (23 mg/g of tissue) (4). Given that there is virtually no exchange of cholesterol with the peripheral circulation, due to the impermeable nature of the blood–brain barrier (BBB), cerebral cholesterol level is dependent on *de novo* synthesis by glial cells (5). However, neurodegenerative diseases as devastating as Niemann-Pick type C (NPC), where the gene affected codes for a lipid transporter in charge of intracellular cholesterol trafficking, evidence the crucial role of this type of lipid manager in brain function.

Therefore, non-enzymatic lipid-interacting proteins are required to carry out a varied set of functions as lipids need to be transported not only between different tissue compartments (outside vs. inside the brain) or between cells (glia-to-glia, glia-to-neurons), but also within cells, across the cytoplasm toward organelles or the nucleus. Other examples include apolipoprotein E (ApoE) carrying lipids between cells, or fatty acid-binding proteins (FABPs), transporting lipids within the cytoplasm. Some carry lipids to the nucleus (retinoic acid receptors; RXRs). Other proteins bind lipids in membranes and make them accessible to enzymes (saposin B) while some are part of complex lipoprotein particles that transport lipids along separate body compartments (high-density lipoprotein; HDL, low-density lipoprotein; LDL). Finally, other transfer lipids between different classes of lipoprotein particles (phospholipid transfer protein; PLTP).

A literature search using a systematic approach helped us to identify (i) key biological processes essential for nervous system function in which lipid management is required, (ii) a set of non-enzymatic lipid-interacting proteins involved in those processes, and (iii) their relationship to neurodegenerative diseases of diverse etiology. The initial search was then combined with process, protein, or disease-specific searches. Table 1 summarizes the findings. We have grouped the biological processes in five major categories (sections Lipid Transfer Across the BBB and Between Cells in the Nervous System to Lipoproteins Involved in Myelin Management), and reviewed the knowledge accrued about the lipid-binding proteins involved. Finally, neurodegenerative diseases recovered in our search (through the published work devoted to non-enzymatic lipid-binding proteins) are discussed (sections Alzheimer's Disease and Cerebrovascular Dementias: Dealing With Amyloid Deposition in a Compromised BBB State to Lysosomal Storage Diseases and Their Inseparable Companions: Leukodystrophies). Our aim is to promote new views in the understanding of neurodegeneration that might seed ideas for potential clinical interventions.

**Table 1:** Non-enzymatic lipid-binding proteins and their involvement in biological processes underlying neurodegenerative diseases.

**Lipid-binding protein**	**Biological process**	**Lipid binding**	**Neurodegenerative disease**
**LIPOPROTEIN PARTICLE SCAFFOLDING PROTEINS AND TRANSFER PROTEINS**			
ApoA-I	Blood–brain barrier function, oxidative stress/inflammation, amyloidogenesis/Aβ clearance	Cholesterol, phospholipids, triglycerides, membranes	Alzheimer's, multiple sclerosis, cerebral amyloid angiopathy, Parkinson's
ApoB100	Blood–brain barrier function	Cholesterol, phospholipids, triglycerides, docosahexaenoic acid	Cerebral amyloid angiopathy, multiple sclerosis.
PLTP	Blood–brain barrier function	Cholesterol, phospholipids, triglycerides	
**MEMBRANE TRANSPORTERS AND RECEPTORS**			
ABCA1	Blood–brain barrier function, HDL biogenesis	Cholesterol, phospholipids, triglycerides	Alzheimer's
ABCG1	Blood–brain barrier function, HDL biogenesis	Cholesterol, phospholipids, triglycerides	
SR-B1	Blood–brain barrier function, HDL biogenesis	Cholesterol, phospholipids, triglycerides	
LDLR	Blood–brain barrier function, amyloidogenesis/Aβ clearance	Lipoprotein particles	Alzheimer's
LRP1	Blood–brain barrier function, Amyloidogenesis/Aβ clearance	Lipoprotein particles	Alzheimer's
Megalin/LRP2	Blood–brain barrier function, amyloidogenesis/Aβ clearance	ApoJ-containing lipoprotein particles	Alzheimer's
S1PR	Blood–brain barrier function, amyloidogenesis	ApoM-containing lipoprotein particles	Cerebral amyloid angiopathy
LSR	Blood–brain barrier function	24(S)-hydroxycholesterol	Alzheimer's
MCT1	Blood–brain barrier function	Ketone bodies	Alzheimer's
**LIPOPROTEIN PARTICLE-ASSOCIATED PROTEINS AND OTHER EXTRACELLULAR LIPID TRANSPORTERS**			
ApoJ/Clusterin	Blood–brain barrier function, oxidative stress/inflammation, amyloidogenesis	Cholesterol	Alzheimer's
ApoE	Blood–brain barrier function, oxidative stress/inflammation, amyloidogenesis	Cholesterol	Alzheimer's, Parkinson's
ApoM	Blood–brain barrier function	Sphingosine-1-P	Vascular dementia
ApoD	Oxidative stress/inflammation, lysosome, myelin	Fatty acids (arachidonic acid), sphingomyelin, anandamide, lysophosphatidylcholine, membranes	Alzheimer's, Parkinson's, multiple sclrerosis, spinocerebellar ataxia 1, lysosomal storage diseases (Niemann–Pick type C)
Lcn2	Oxidative stress/inflammation, lysosome, myelin	Bacterial siderophores	Fabri's disease, neuronal cerois lipofuscinosis
**INTRACELLULAR LIPID-BINDING PROTEINS**			
FABP7/B-FABP	Cytoplasmic lipid transport	Fatty acids, retinoids, eicosanoids	Alzheimer's, Parkinson's, Down syndrome
FABP3/H-FABP	Cytoplasmic lipid transport	Fatty acids, retinoids, eicosanoids	Alzheimer's, Parkinson's, Lewy body, vascular dementia, Creutzfeldt–Jakob
FABP5	Cytoplasmic lipid transport	Fatty acids (docosahexaenoic acid)	Alzheimer's
Alfa-synuclein	Synaptic vesicle	Membranes (acidic phospholipids)	Alzheimer's, Parkinson's
NPC1 and NPC2	Lysosome, myelin	Cholesterol	Lysosomal storage diseases (Niemann–Pick type C)
Saposin B	Lysosome, myelin	Membranes (glycosphingolipids)	Lysosomal storage diseases (metachromatic leukodystrophy)
Hsp70	Lysosome	Membranes (phospholipids)	Lysosomal storage diseases (Niemann–Pick type A)
**LIPID BINDING TRANSCRIPTION REGULATORS**			
RXR	Transcription regulation	Retinoic acid	
LXR	Transcription regulation	24(S)-hydroxycholesterol	

*Lipid-binding proteins are grouped by type of lipid management and are assigned one or various of the five general biological processes reviewed: (1) blood–brain barrier function (including transport across and functional maintenance), (2) control of oxidative stress and inflammation, (3) Aβ dynamics (amyloidogenesis or Aβ clearance), (4) organelle membrane-related functions (including lysosomal and synaptic vesicle functions), and (5) myelin management function. Major lipid ligands for each protein are listed. Neurodegenerative diseases that are directly related or modified by the function of the lipid-manager proteins are listed*.

## Lipids and Their Protein Managers Are Involved in Key Processes Essential for a Healthy Brain

### Lipid Transfer Across the BBB and Between Cells in the Nervous System

Our brain constitutes a separate compartment for lipid management due to the highly controlled BBB. Brain capillary endothelial cells constitute the barrier itself, with tight junctions blocking the passage of substances across the capillary walls, and a set of specific receptors and transporters controlling the traffic across them. Astrocytes and pericytes help in the development and maintenance of the barrier through constant exchange of intercellular signaling that conditions endothelial gene expression and ultimately results in BBB permeability properties. They also modulate the availability of substances crossing the BBB by their effect on activity-dependent blood flow control (6, 7).

**Figure 1:**
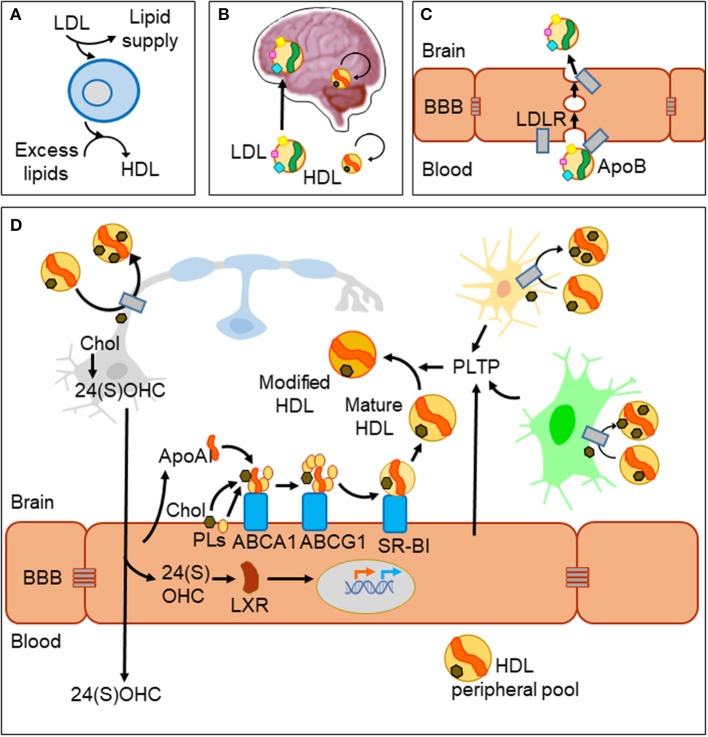
Lipid management in the brain and across the blood–brain barrier. **(A)** Two major classes of lipoprotein particles are in charge of providing lipids to cells (LDL) and removing excess lipids (HDL) to fulfill cellular demands. **(B)** The blood–brain barrier (BBB) imposes limitations to traffic from the circulation to the brain parenchyma. LDL particles originated outside the brain cross the BBB, while biogenesis of HDLs occurs in two separate pools. HDLs cross the barrier only under conditions of damaged BBB. **(C)** LDL uses a receptor-mediated transcytosis mechanism to cross BBB endothelial cells. **(D)** HDL biogenesis and cholesterol recycling within the brain. HDL scaffolding protein ApoA-I and its receptors are expressed by endothelial cells and biogenesis takes place at the brain side of the BBB. Nascent HDLs are then modified by lipid transfer proteins (PLTP) secreted from endothelial and glial cells. HDL particles have receptors in all cell types of the nervous system where they uptake excess lipids (cholesterol and others). Under normal conditions, HDLs exit the brain through bulk flow from the extracellular space to the circulation and lymphatic systems. Part of cholesterol recycling takes place thanks to the production of 24(S)-hydroxycholesterol by the neuronal-specific CYP46A1 enzyme. 24(S)OHC freely crosses the BBB and is also able to bind LXR proteins in the cytoplasm of endothelial cells, which translocate to the nucleus and promote the transcription of ApoA-I and the receptors involved in HDL biogenesis.

In spite of the BBB, metabolic diseases altering systemic lipid profiles clearly affect brain function and homeostasis. A well-documented example is the inverse relationship between levels of cholesterol in plasma high-density lipoprotein (HDL-C) and risk of Alzheimer's disease (AD) and other dementias (8). Lipoprotein particles are emulsions of metabolites, lipids, proteins, and microRNAs (9). They are classified in two major types (HDL and LDL) that differ in their density and lipid content, the main protein serving as scaffold (apolipoprotein A-I, ApoA-I in HDL; apolipoprotein B, ApoB in LDL) and their lipid transport effect (removing excess lipids from cells or supplying lipids to cells, respectively, Figure 1A). HDL-like particles are formed in the brain compartment as a separate pool independent of plasma HDL, and they do not cross the BBB under normal conditions (Figure 1B). By contrast, LDL biogenesis occurs outside the brain, and LDL receptors (LDLR) mediate its passage from blood to brain by transcytosis (10) (Figure 1C). Various members of the LDLR family are expressed by glial cells and neurons as well. However, we must take into account that despite their name, these receptor proteins have a wide array of ligands and functions and are not limited to the classical ApoB-containing LDL particles (11). As an example, the LDL receptor-related protein 1 (LRP1), a membrane-associated protein highly expressed by BBB endothelial cells, has been described as the major pump responsible for the efflux of amyloid-β (Aβ) out of the brain (12).

Acting in conjunction with HDL particles, a panel of lipid-interacting proteins are expressed by brain endothelial cells, contributing to cholesterol recycling within the brain and trafficking excess cholesterol out of the brain (13, 14) (Figure 1D). HDL biogenesis takes place at the basolateral side (“brain-side”) of BBB endothelial cells. They express ApoA-I (15, 16), contributing to the CNS pool of this apolipoprotein, one of the most abundant in the cerebrospinal fluid (CSF) (17). BBB endothelial cells also express the set of membrane proteins required to initiate ApoA-I uptake of lipids (ABCA1; ATP-binding cassette transporter A1), and to complete its maturation (ABCG1 and SR-BI; scavenger receptor B1), all of them lipid-interacting proteins transferring cholesterol and phospholipids from the cell to the nascent HDL particle (9). Other lipid-binding proteins are involved in remodeling HDL particles, like PLTP (secreted from endothelial and glial cells), giving rise to pre-β-HDL and HDL_2_, HDL forms particularly suitable as acceptors of excess cellular cholesterol (13). As a parallel mechanism, a brain-specific enzyme (CYP46A1) converts excess cholesterol to 24(S)OHC [24(S)-hydroxycholesterol] in neurons with two effects: (1) It can readily cross the BBB and activate nuclear liver-X-receptor (LXR) transcription factors within endothelial cells. (2) LXRs turn on the expression of proteins involved in HDL biogenesis mentioned above (14) thus resulting in cholesterol turnover. Yet another lipoprotein receptor plays a role in cholesterol exchange: the lipolysis-stimulated lipoprotein receptor (LSR). A reduced LSR expression in aged mice results in higher cortex levels of 24(S)OHC (18). This constitutes an example of an indirect mechanism by which alterations in an LDL receptor impairs efflux of brain cholesterol, affecting the cholesterol turnover rate.

Some lipid-binding proteins associated with HDL-like lipoprotein particles can be conceptualized as chaperones of particular lipids that define particle sub-types. ApoJ/Clusterin, ApoE, or ApoM are good examples of this class of proteins. In spite of their name, they belong to different protein families, and bind lipids in a different way. They contribute to modulate not only the lipid cargo of HDLs but also their docking at specific cell surface receptors. ApoJ binds cholesterol and uses megalin/LRP2 as receptor (17, 19, 20). ApoE binds cholesterol and uses preferentially LRP1 receptor (21). Finally, ApoM binds specifically to sphingosine-1-P (S1P) and uses the receptor S1PR to dock ApoM-HDL particles to the receiving cell surface (22, 23).

Protein carriers transporting single lipid molecules also mediate lipid transport across BBB endothelial cells. An example of this is the monocarboxylic acid transporter (MCT1), expressed by endothelial cells and also by glial cells within the brain. MCT1 transports ketone bodies, originated by liver metabolism, across the BBB (24). This process takes place in conditions of diminished glucose availability, when the brain undergoes a metabolic switch toward the utilization of fatty acids as energetic fuel (25).

Extracellular lipocalins and cytoplasmic fatty acid-binding proteins (FABPs) are single-domain small proteins with a β-barrel structure (of 8 and 10 anti-parallel β-strands, respectively) that holds a ligand-binding pocket. Brain expression is found for various members of the lipocalin family, like apolipoprotein D (ApoD), mainly in myelinating glia and reactive astrocytes, lipocalin-type prostaglandin D synthase (LPGDS), in oligodendrocytes, or lipocalin 2 (Lcn2), in reactive astrocytes (26). On the other hand, cytoplasmic FABPs like FABP3/H-FABP or FABP7/B-FABP (the later uniquely expressed in astrocytes) or FABP5 (expressed by BBB endothelial cells) are also brain-born. They can be described as “chaperones” that facilitate the transport of small hydrophobic ligands [retinoic acid (RA), docosahexaenoic acid (DHA), arachidonic acid (AA), or endocannabinoids like arachidonoylethanolamide (AEA) and 2-arachidonoyl-glycerol (2-AG)] (27). For example, DHA, an essential omega-3 long-chain polyunsaturated fatty acid, is known to have anti-inflammatory properties on glial cells and influences memory and cognitive functions (28). After its transport in blood via the lipocalin retinol-binding protein 4 (RBP4), LDL lipoparticles, or serum albumin (29), it crosses the cell membrane and travels across the brain endothelial cell cytoplasm via FABP5 (30). Lipocalins and FABPs manage lipids not only across the BBB but also between cells in the nervous system (glia-to-glia or glia-to-neuron). This system can be visualized as an extracellular–intracellular relay race, serving intercellular exchange of lipid-mediated signaling, whose disruption contributes to many neurodegenerative conditions.

### Lipoproteins Keeping Oxidative Stress and Inflammation Under Control

As depicted above, carrying lipids from one compartment to another or from one cell to another uses diverse systems, with lipids managed either in lipoprotein particles or by single domain small protein carriers. To this complexity, we have to add that lipoprotein particles, lipocalins, and cellular FABPs are not mere lipid transporters, but execute other functions that are especially relevant in the context of nervous system homeostasis: they contribute to control oxidative and inflammatory states.

Oxidative stress (OS) is an imbalance between the production of reactive oxygen species (ROS) due to aerobic metabolism in mitochondria and the antioxidant defenses that counteract them. When ROS are not properly neutralized by antioxidants, they can oxidize DNA, lipids, and proteins, altering their normal function. OS is also implicated in many neurodegenerative diseases, including Parkinson's disease (PD) (31) and AD (32), and is a landmark of physiological aging (33). ApoD is one of the most consistently upregulated proteins in the mammalian aging brain (34), a process highly related to OS production and deficient antioxidant capabilities. While in normal conditions ApoD is expressed at low levels by astrocytes and myelinating glia, its expression is quickly increased upon a neural insult due to trauma, exposure to exogenous toxics, or a wide array of neurodegenerative processes (35). Aging and disease-triggered overexpression of ApoD constitutes an endogenous mechanism of protection, as demonstrated by the mirror effects of the OS-generator Paraquat in loss-of-function and gain-of-function ApoD mutants in *Drosophila* and mouse (36–39). ApoD absence results in a pro-oxidative state in the brain, specifically altering lipid peroxidation (37) and resulting in an accelerated aging of brain functions (40). A mechanism of inhibition of lipid peroxidation has been demonstrated for human ApoD, which is able to reduce lipid hydroperoxides (LOOHs) to inert lipid hydroxides (LOHs) thanks to a particular methionine residue (41). It is interesting to note that one of the mechanisms behind the antioxidant properties of ApoA-I in HDL particles is also based on a methionine-dependent reducing activity on LDL-associated lipids (9). ApoA-I also helps to keep LDL particles resistant to oxidation by removing lipids prone to oxidation. These ApoA-I antioxidative functions counteract the deleterious effect of oxLDL particles on the BBB and contribute to keep HDL lipids in their reduced form. Finally, ApoJ/Clusterin is also thought to be a sensitive biosensor of OS. Its promoter has some OS-responsive binding sites (42), and ApoJ has been postulated as a protective molecule against OS based on gene silencing experiments (43). In addition, ApoJ also contributes to manage inflammation through binding to misfolded proteins and peptides, an interesting aspect shared by various lipid-binding proteins (see below).

In addition to OS, inflammation is a common pathophysiological factor present in the aging brain and neurodegenerative disorders. During aging, the organism suffers an imbalance between inflammatory and anti-inflammatory pathways, rendering a state of low-grade chronic inflammatory status known as “inflammaging.” This status is characterized by a higher propensity to inflammation and a lower efficiency of inflammatory responses (44). Lipoproteins are in fact able to modulate polarization of macrophages and microglia in the anti/pro-inflammatory axis. For example, ApoE-LRP1 interaction mediates downregulation of microglial pro-inflammatory activity, by reducing JNK pathway activity (45). Also, ApoE induces polarization of macrophages to the M2 anti-inflammatory phenotype upon binding to LRP1 or ApoER2 receptors (46). These effects are relevant in disease conditions, where macrophages infiltrate the nervous system parenchyma or generate pro-inflammatory mediators (like TNF-α) (47) that cross the BBB and alter basal neuroinflammatory state. In addition, HDLs inhibit cytokine-induced expression of adhesion molecules in endothelial cells (48), which would restrain the macrophage–endothelial cell contacts required for access into the brain, and thus would hold back neuroinflammation. The lipocalin ApoD is also known to have anti-inflammatory functions (49, 50). ApoD controls the extent and duration of the inflammatory response upon peripheral nerve injury, by binding lysophosphatidylcholine (LPC) and arachidonic acid (AA) and dampening the Schwann cell signaling that recruits and activates macrophages.

In summary, lipid-binding proteins not only contribute to keep an adequate redox state in the healthy brain by mechanisms directly related to their lipid management properties but also contribute to the degree and duration of cellular responses to brain injury and disease by influencing pro- or anti-inflammatory signaling cascades.

### Lipid-Binding Proteins' Influence on Amyloidogenesis

Misfolded proteins and toxic peptides are a primary cause in many neurodegenerative diseases. Curiously, these toxic peptides are often hydrophobic (hence their tendency to form aggregates) and can be bound by lipid-binding proteins or lipoprotein particles (Aβ being a well-known example). Therefore, because of shared biophysical properties, lipid-binding proteins are also responsible for managing hydrophobic peptides, like Aβ, in the brain. In other instances, the lipid-binding moiety of the protein can become susceptible of aggregation, as in α-synuclein (see below), becoming part of the pathogenic mechanism.

**Figure 2:**
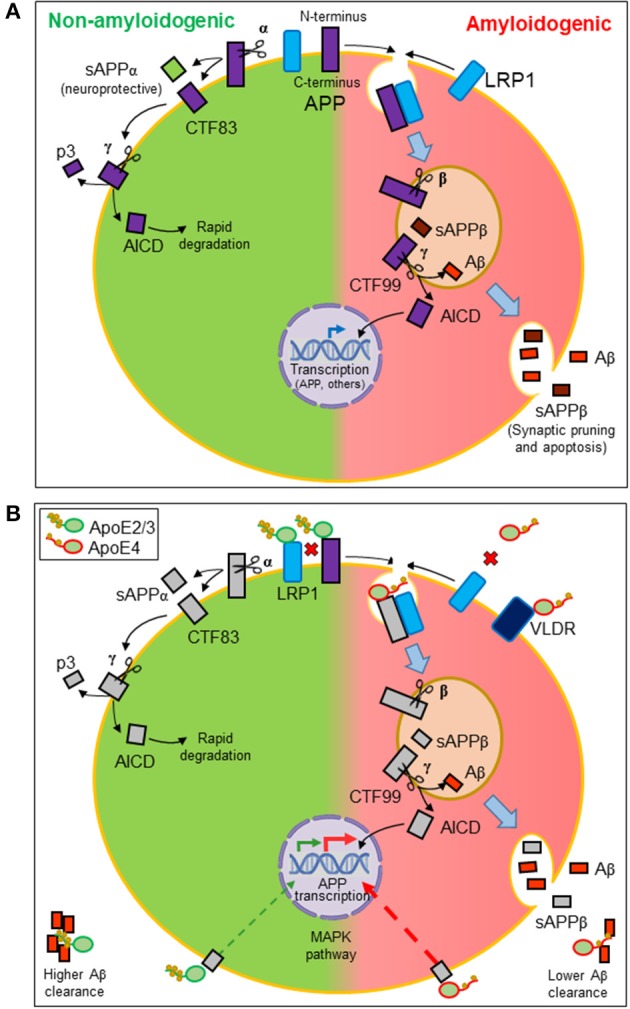
Amyloidogenesis and the influence of lipid managing proteins. **(A)** Alternative proteolytic processing of amyloid precursor protein (APP). Plasma membrane α-secretase initiates the non-amyloidogenic pathway, releasing a soluble ectodomain (sAPPα) and generating a membrane-anchored C-terminal fragment (CTF) of 83 amino acids. In contrast, the amyloidogenic pathway is initiated by β-secretase in intracellular acidic organelles. Its processing results in a 99-amino-acid CTF and a soluble extracellular sAPPβ. CTFs are subsequently processed by γ-secretases generating p3 or Aβ peptides and releasing an APP intracellular domain (AICD). The AICD resulting from the amyloidogenic pathway translocates to the nucleus and regulates amyloidogenic and lipid metabolism gene transcription. The AICD generated by the non-amyloidogenic pathway is rapidly degraded in the cytosol. **(B)** Effects of ApoE on the production and dynamics of Aβ peptides. Direct interaction of ApoE with APP modulates its internalization. Also, LRP1 receptor promotes APP internalization. Because ApoE2/ApoE3 isoforms have more affinity for this receptor than ApoE4, ApoE2, and ApoE3 avoid APP internalization, preventing the amyloidogenic pathway (disfavored interactions depicted as red crosses). Moreover, ApoE triggers a signaling pathway that stimulates APP transcription. ApoE4 is the most potent isoform in triggering this pathway and thus Aβ production. Finally, ApoE also binds hydrophobic Aβ peptides through its lipid-binding domain. The lower lipid load of ApoE4 isoform results in a lower efficiency in Aβ clearance.

Aβ peptides originate from the proteolytic processing of amyloid precursor protein (APP), a ubiquitously expressed type I transmembrane protein that traffics between the plasma membrane and acidic intracellular compartments (5). Figure 2A summarizes the two alternative APP processing pathways. The non-amyloidogenic pathway is predominant in the healthy brain and takes place at the cell surface, where the α-secretase initiates APP processing. The amyloidogenic pathway, which results in the synthesis of Aβ peptides, requires internalization of APP into acidic compartments where β-secretase is at its optimal pH. In both pathways, membrane-anchored C-terminal fragments (CTFs) are subsequently processed by γ-secretase to generate p3 or Aβ peptides, concomitantly with the release of an APP intracellular domain (AICD) into the cytosol. APP metabolites play their own roles in brain function: sAPPβ seems to be involved in synaptic pruning and apoptosis (51), while sAPPα is considered neuroprotective (52). The AICD resulting from the amyloidogenic pathway is known to translocate to the nucleus and regulate gene transcription, with APP, β-secretase, neprilisin, and several enzymes involved in lipid metabolism as target genes. In contrast, the AICD generated by the non-amyloidogenic pathway is rapidly degraded in the cytosol (5).

Brain HDL scaffolding proteins like ApoA-I, or associated proteins like ApoJ, have been demonstrated to impact Aβ-related pathogenesis, by influencing its oligomerization, its traffic through the BBB (in or out of the brain), as well as its endocytosis and degradation by cells within the brain or at the vascular endothelium. ApoA-I is able to bind soluble Aβ (53), and this interaction is thought to facilitate Aβ efflux from the brain through bulk flow into the CSF, from which it would be transferred to the circulatory and lymphatic systems. Clearance will avoid Aβ oligomerization and toxicity within the parenchyma. ApoJ is also able to interact with Aβ, apparently altering amyloid aggregation and promoting Aβ clearance (54). However, the specific role that ApoJ plays on Aβ-induced pathology remains unclear. It has been proposed that Aβ-ApoJ interaction is dependent on the ApoJ:Aβ ratio, which could determine whether ApoJ exhibits neuroprotective or neurotoxic effects (55). Endocytosis of Aβ-ApoJ complexes is mediated by interaction with megalin/LRP2 receptor at the BBB endothelial cells (19).

ApoE is highly expressed in the CNS, with no exchange of brain-derived and peripheral ApoE, therefore constituting two independent pools (21, 56, 57). Astrocytes are the primary source of ApoE in the brain (58–60), and it is also expressed at a lesser extent by microglia (61). ApoE plays a major role in cholesterol and phospholipid management within the CNS, and uses preferentially the LRP1 receptor (21). The primary function of ApoE is the maintenance of specific lipoprotein particles structure and docking them to specific cell receptors, carrying out lipid transport not only between cells in the nervous system but also across a defective BBB in pathological conditions (57).

ApoE takes part in HDL-like particles biogenesis, by interaction with ABCA1 receptors, which leads to varied degrees of lipid uptake by ApoE (62, 63). Its C-terminal domain constitutes its lipid-binding moiety, while the N-terminal one binds to receptors. The three most frequent alleles in humans (originating protein isoforms ApoE2, ApoE3, and ApoE4) have variations in amino acids in the receptor-binding domain (21). These ApoE isoforms present differential affinities for lipoprotein receptors located at the BBB and neuronal membranes, with ApoE2 and ApoE3 being the main interactors with LRP1, while ApoE4 binds preferentially to very-low-density lipoprotein receptor (VLDR) (60). The differential interaction with receptors results in differences of lipid-binding capacity for the different ApoE isoforms. ApoE4 is thought to have a molten globule structure, different to the structure of the other two isoforms (64), which makes it less stable, more easily degraded by astrocytes, with a lower level of lipid-loading capacity, and less affinity for LDLR family receptors and for APP (65).

With these properties, ApoE participates in various processes related to APP processing (Figure 2B). On the one hand, ApoE direct interaction with APP (66) regulates its internalization and the amyloidogenic pathway. On the other hand, LRP1 accelerates the endocytic traffic of APP, therefore promoting amyloidogenesis (67). We can define these relationships as competitive interactions. When ApoE2 or ApoE3 isoforms are expressed, HDL-ApoE performs its normal lipid transport, docking into the LRP1 receptors in neurons or endothelial cells while preventing APP to be internalized and processed through the amyloidogenic pathway. The lower lipid uptake capacity of ApoE4 and its lower affinity for LRP1 and APP has two consequences: (1) a less efficient intercellular lipid transport, and (2) a higher probability of LRP1-APP interaction, mostly in neuronal membranes, initiating APP internalization and the amyloidogenesis pathway (21, 57). Additionally, ApoE is able to trigger a non-canonical MAP kinase pathway that stimulates APP gene transcription, with ApoE4 being the isoform showing the greatest potency in such stimulation, thus promoting Aβ production further (68).

In addition to the effects on Aβ production, ApoE also binds hydrophobic Aβ peptides through its lipid-binding domain, an interaction that again depends on the lipid load of each isoform. This binding is also pH-sensitive (57), making the interaction to be location-dependent (intracellular acidic organelles vs. neutral extracellular space). Moreover, ApoE4 shows less affinity for Aβ peptides, making it a less efficient apolipoprotein in Aβ clearance from brain to blood (69). In summary, ApoE4 isoform is less suited for its intercellular lipid management function: ApoE4 uptakes a smaller lipid load at origin, and binds poorly to LRP1 at destination. Furthermore, during its traffic between cells, ApoE4 is a poor Aβ acceptor, and at destination, it promotes yet another deleterious effect: amyloidogenesis.

An important aspect of the just discussed effects of lipid-managing proteins on the production and dynamics of Aβ or other misfolded proteins in the extracellular space is that, consequently, inflammatory responses are inhibited. Thus, lipid-binding proteins also contribute to keep neuroinflammation under control not only because of their signaling properties mentioned above but also as a side effect of their ability to manage hydrophobic toxic peptides like Aβ.

### Lipid-Binding Proteins Taking Care of Organelle Membranes and Proper Vesicle Trafficking

To the transport of lipids across water-based fluids, we have to add lipid management and interactions within biological membranes as a second major role for non-enzymatic lipid-binding proteins. We will focus next on three biological membranes that have special interest for the healthy and diseased nervous system: synaptic vesicles, lysosomes, and myelin.

#### Synaptic Vesicle Management

α-Synuclein is located in the cytoplasm of neurons and glial cells, but is known to interact with membranes through acidic phospholipids (70). α-Synuclein–membrane interactions have gained recent attention, with data accumulating on its function as regulator of membrane lipid composition, with putative roles in fatty acid traffic within the cell (71). Within neurons, α-synuclein is enriched in synaptic terminals, where it serves important functions in synaptic vesicle traffic (72), participating in the vesicle docking system at the presynaptic terminals, as well as in clathrin-dependent vesicle recycling essential for proper neuronal function (73). The fact that mutations in the α-synuclein locus cause familial forms of PD has triggered much research on its biochemical and biophysical properties. In this context, a curious property is shared between some apolipoproteins like ApoA-I and α-synuclein. They contain 11 amino acid repeats that form amphipathic helices that allow these proteins to be inserted into cell membranes. Thanks to this interaction both lipid-binding proteins are able to modulate membrane curvature (74). Thus, a common mechanism is used by ApoA-I/ABCA1 complexes to initiate lipid uptake by ApoA-I and HDL biogenesis (9), and by the α-synuclein for its membrane interaction.

#### Securing Lysosomal Function

Stability of lysosomal membrane is crucial for cellular homeostasis and becomes a strategic factor for cell survival or death (75). Many proteolytic and lipolytic enzymes can only work properly within a well-controlled lysosome, able to maintain a luminal pH lower than the surrounding cytoplasm. In addition to those with enzymatic activity, various lipid-binding proteins exert functions within the lysosome. Saposin B works as glycosphingolipid presenter for lysosomal enzymes (76). Others regulate lipid-modifying enzymes, like Hsp70, whose binding to endolysosomal phospholipids regulates the activity of acid sphingomyelinase (ASM) (77). Furthermore, a healthy lysosome is required for a proper autophagic flux, which conditions the clearance of aggregation-prone proteins and other deleterious materials invariably produced in cells along their lifetime. Neurons are particularly vulnerable to autophagy disruption, since they are post-mitotic long-living cells. Lysosomes are also involved in the degradation of exogenous material by phagocytosis, a process particularly important after nervous system injury or upon demyelinating conditions, where myelin debris have to be efficiently removed if neuronal regeneration is to take place. Therefore, the composition of lysosomal membranes is crucial for both types of lysosomal function: (1) it has an impact on its permeability properties, and thus the maintenance of an intra-lysosomal milieu optimal for the enzymatic activities; (2) it conditions the association of particular proteins and chaperones to the lysosomal membrane and its fusion to autophagosomes or phagosome membranes.

A somehow surprising finding is that lipid-binding proteins, classically viewed as lipid transporters across extracellular fluids, have also essential roles in managing lysosomal membrane stability. That is the case of the lipocalin ApoD in both glial cells that express it (astrocytes and oligodendrocytes) and neurons (in a paracrine manner) (39, 78). As stated above, ApoD prevents lipid peroxidation, and its presence in lysosomes is required to avoid lysosomal permeabilization in oxidation-promoting situations. In OS conditions ApoD traffics from plasma membrane to lysosomes and guarantees that optimal pH for lysosomal enzymes is kept under control (78). In addition, Glial Lazarillo, a close homolog of ApoD in Drosophila, is required for proper lysosome–autophagosome fusion, which maintains an adequate autophagy flux in a proteinopathic condition (Type I Spinocerebellar Ataxia, SCA1) (79). Other lipocalins like Lcn2 have also been implicated in lysosomal membrane management. Curiously, Lcn2 promotes lysosomal permeabilization, inhibiting autophagic flux in cardiomiocytes (80) and promoting apoptotic cell death (81). This Lcn2 function, discovered outside the nervous system, also takes place in the nervous system cells expressing Lcn2 (astrocytes) or receiving it (neurons, microglia, and oligodendrocytes). Reactive astrocytes induce Lcn2 expression in response to inflammatory conditions and induce their own cell death as well as neuronal death (82). Neurodegenerative conditions of diverse etiology trigger the expression of Lcn2 and ApoD (35, 83). Therefore, these two lipid-binding proteins of the Lipocalin family seem to represent the opposite sides of the important equilibrium between neurodegeneration and neuroprotection, with the control of lysosomal functions as the center stage.

### Lipoproteins Involved in Myelin Management

Although not previously anticipated, lysosomal stability and proper lysosomal function are also essential for adequate myelin maturation and for the physiological constant recycling of myelin membranes. Myelin is a very specialized type of plasma membrane assembled by specific glial cells in both CNS and PNS. Extensive membrane growth and recycling through endosomal and lysosomal pathways take place during myelin biogenesis (84), and remodeling continues through adulthood in an activity-dependent manner (85). Myelin properties are essential in determining the conduction velocity and thus neural circuit performance. Altered myelin function clearly leads to behavioral and cognitive deficits (86). Lysosome–myelin relationship is based on the fact that many of the lipidic components of myelin (accounting for 70–80% of its dry weight) are produced or recycled in lysosomes. The lipocalin ApoD was recently shown to be essential for the completion of the myelin compaction process (87). Without ApoD, myelin membrane recycling through the lysosome is altered, leading to lipid composition changes. An excess of glycosylated lipids (gangliosides) prevents the compaction of the extracellular leaflet, resulting in a less hydrophobic myelin. As a result, nerve conduction velocity is reduced (49).

In addition to the lipid management required during myelin construction and remodeling, myelin catabolism represents another process where proper lipid-binding proteins must be necessary, in addition to the involved enzymes. Myelin catabolism in the aging female brain has been proposed as an adaptive response (88). White matter degeneration generates ketone bodies as part of a shift in brain bioenergetics. As a result, levels of ceramides and various fatty acids (DHA, AA, palmitic, and oleic acids) increase, posing an inflammatory challenge to the aging brain. It is therefore worthwhile to analyze how myelin catabolism-derived lipids are managed during physiological or pathological aging. Upon nervous system injury or in demyelinating diseases, myelin debris are phagocytosed either by resident microglia or Schwann cells or by infiltrating macrophages. Again, in those processes, lipid-binding and membrane-interacting lipoproteins are required. In these situations, ApoD and Lcn2 also play opposite roles, with ApoD favoring myelin phagocytosis and removal (50, 87) and Lcn2 promoting demyelination (89).

## Neurodegeneration Understood From a Lipid Manager Point of View

### Alzheimer's Disease and Cerebrovascular Dementias: Dealing With Amyloid Deposition in a Compromised BBB State

Alzheimer's disease is the most common form of dementia, characterized by deposition of amyloid plaques in brain extracellular space and neurofibrillary tangles inside neurons. Aβ toxic oligomers cause synaptic dysfunction and a series of downstream events, ultimately leading to neuronal cell death. The majority of AD cases are sporadic with a late onset and do not have strong genetic components as the primary cause. Hence, we need to understand all putative risk factors in order to deal with this excruciating health problem. As mentioned above, the best known and objective risk factor for sporadic AD is the ApoE4 allele, which brings lipid-binding proteins up front in the management of the lipophilic Aβ peptides.

Once Aβ is generated by amyloidogenic processing of APP (see above; Figure 2), it is released to the interstitial fluid where clearance takes place by different mechanisms: efflux across BBB via ApoE receptors (mainly LRP1 and VLDLR), uptake by cells for lysosomal degradation, and cleavage by Aβ-specific proteases. As reviewed above, members of the LDLR gene family have a broad set of biological functions beyond lipid metabolism. LRP1 interactions with either APP or lipid-loaded ApoE in the cell membrane promote or discourage APP internalization, and therefore amyloidogenesis (67), in an isoform-dependent manner (Figure 2B). LRAD3, a newly identified member of the LDLR family, also binds APP and promotes its amyloidogenic processing (90). LRP1B has a slower rate of endocytosis and retains APP at the cell surface (91). Finally, LRP10 binds to APP and promotes its traffic to the Golgi complex, which also prevents amyloidogenesis (11). LRP1, expressed in neurons, influences not only amyloidogenesis but also clearance of extracellular Aβ by promoting its uptake and degradation (92). A similar Aβ clearance mechanism, though taking place in astrocytes and microglia, has been described for ApoJ–Aβ complexes that use the Megalin/LRP2 receptor instead (93, 94). Yet, an additional effect of Aβ is its ability to be inserted in brain cell membranes, altering membrane fluidity and initiating a lipid peroxidation chain reaction. Among the multiple consequences of the OS generated, lipid peroxidation results in the addition of 4HNE adducts to LRP1. The oxidized LRP1 is less active in clearing ApoE–Aβ complexes and, in turn, causes more accumulation of Aβ in the AD brain (12), thus generating a negative synergy between OS and Aβ management.

The majority of AD patients have co-morbid vascular diseases and cerebrovascular pathologies. Most also have Aβ deposition in cerebral arteries, known as cerebral amyloid angiopathy (CAA) (8). Therefore, lipid-binding proteins that influence the cerebrovascular system integrity and control Aβ clearance across the BBB become relevant targets toward treating AD and vascular dementias in general. Leakage of molecules across the BBB could be caused by loss or defective tight junction proteins between endothelial cells and/or enhanced transcytosis across the BBB (95). For example, the CSF of AD patients shows low levels of the Lipocalin ApoM (96). ApoM binds S1P in the blood and carries it to the membrane receptor S1PR in endothelial cells, where S1P promotes their barrier properties, preventing leakage and subsequent neuroinflammation. S1PR activation also decreases the expression of adhesion molecules used by leukocytes to adhere to endothelial cells before infiltration into damaged tissues (23). Therefore, a dysfunction of this ApoM-dependent mechanism might contribute to the BBB breakdown observed in AD patients. ApoA-I is also a relevant player at the cerebrovasculature. ApoA-I originated outside the brain acts from the lumen of blood vessels, precluding deposition of amyloid at the blood side of the BBB, thus preventing amyloid angiopathy and BBB disruption (97). ApoA-I deficiency in APP/PS1 mice increases CAA, total cortical Aβ deposition, and several markers of neuroinflammation (8).

Yet, other factors might influence Aβ clearance at the BBB, early in the disease progression. Multiple studies have shown impaired regional brain uptake of glucose before neurodegeneration, suggesting reduced glucose brain utilization caused by decreased glucose transport across the BBB via endothelial-specific glucose transporter GLUT1 (98). Moreover, diminished GLUT1 expression in brain endothelium leads to transcriptional inhibition of LRP1, accelerating Aβ pathology (99).

In summary, in the brain of AD and related vascular dementias, deleterious effects of amyloid peptides are combined with pro-oxidative and pro-inflammatory conditions in a disrupted BBB situation. Independently of the primary cause of each particular disease, lipid-binding proteins are involved in every step.

### Parkinson's Disease: An Oxidative Challenge for Dopaminergic Neurons

PD is an age-related neurodegenerative disorder characterized by dopaminergic neuronal cell death in the brain substantia nigra. The main triggering factor of the disease are Lewy bodies, intraneuronal protein aggregates composed of α-synuclein (100). Various events associated with PD such as OS, endosomal–lysosomal dysfunction, endoplasmic reticulum stress, and inflammatory responses, have been described as processes in which lipids play a key role (3).

Mutations in α-synuclein are causative for familial PD; thus, PD is considered as one of the synucleinopathies, which include diseases that affect both neurons and oligodendroglia (101). In addition to the functional consequences derived from synaptic malfunction in PD, research aiming at understanding factors triggering α-synuclein aggregation brings its membrane interaction up front. Although some controversy exists about the structure and oligomer conformation of native α-synuclein (102, 103), there is consensus on the concept that α-synuclein interaction with membranes is the key factor conditioning transitions between different structural modes and, therefore, its susceptibility to form pathogenic fibrillar aggregates (70). Factors reducing α-synuclein/membrane interaction promote its aggregation. Furthermore, this membrane interaction is regulated by polyunsaturated phospholipids (PUFAs), the cellular membrane components with the highest susceptibility to oxidative damage. They interact with α-synuclein and stabilize its binding to cell membranes when they are in their reduced state. The OS that develops in dopaminergic neurons of PD patients results in oxidation of PUFAs and oxidative modification of α-synuclein. Both effects reduce the affinity of α-synuclein–lipid interaction and promote the formation of toxic protein oligomers (104).

*In vitro* studies on toxin-mediated proteasomal impairment in the dopaminergic cell line SH-SY5Y have suggested that ApoJ/Clusterin also prevents α-synuclein aggregation due to its chaperone activity (105), probably involving their hydrophobic lipid-binding moieties. Consistent with this idea, Lewy bodies in PD and other synucleopathies show an inverse correlation between ApoJ and α-synuclein content (106). ApoA-I levels also decrease in PD, especially at early stages of the disease. The increase in oxidation markers produced in PD brains concurs with ApoA-I oxidative damage, resulting in malfunction of cholesterol processing, dysregulation of the inflammatory response, and acceleration of neurodegeneration. Among other effects, oxidized ApoA-I loses its ability to inhibit TNF-α release, which is exacerbated by OS and can cross the BBB, thereby leading to neuronal death (97).

Since ApoD possesses neuroprotective effects against OS, and pro-oxidative stimuli regulate its expression in astrocytes (39), upregulation of ApoD in the brain of PD patients is not unexpected. Increase in ApoD immunoreactivity has been in fact observed in glial cells of the substantia nigra (107) and in brainstem neurons and glia of PD patients (108). This upregulation can be interpreted as a protective mechanism against neurodegeneration, where glia-derived ApoD is internalized by stressed neurons (109). This neuroprotective function is supported by the work on model organisms (36, 37). Lack of ApoD in mouse promotes astrogliosis in the substantia nigra and functional alterations in dopaminergic neurons (39).

Finally, cholesterol deficiency in the brain causes impaired neuronal plasticity and reduced neurotransmission. Since ApoE is mainly responsible for the maintenance of cholesterol homeostasis within the CNS, it is plausible that ApoE might also be involved in PD. In fact, ApoE has been proposed as a risk factor for PD, and the three ApoE isoforms might play different roles in the pathogenesis of PD through their differential interaction with LRP1, as it is the case for AD. An *in vitro* study evaluating the effects of ApoE isoforms on α-synuclein aggregation showed that ApoE4 increases aggregation of α-synuclein more than other isoforms (110) in a mechanism resembling Aβ management in AD.

In summary, various lipid-binding proteins, in addition to α-synuclein, are implicated in lipid interactions and management in the altered conditions present in PD. Interestingly, most of them also influence AD and other common neurodegenerative pathologies. Knowledge of their functional connections can definitely help to broaden the strategies to palliate or counteract disease progression.

### Multiple Sclerosis and Other Demyelinating Diseases: BBB and Myelin at Stake

Multiple sclerosis (MS) and other demyelinating diseases are caused by autoimmune attacks toward myelin components in a pro-inflammatory context associated with BBB disruption and infiltration of immune system cells. In these pathologies, the influence of lipid-managing proteins in BBB stability becomes a relevant factor and a process where preventive or repairing interventions would be desirable.

Three ApoM-related processes influence BBB permeability. ApoM and ApoM-positive HDL particles contribute to control neuroinflammation at different levels (23, 111, 112). First, S1P-ApoM complex restrains lymphopoiesis, thus controlling inflammatory state at origin. Second, ApoM brings S1P to brain endothelial cells, influencing their permeability properties through signaling cascades initiated by S1PR binding. Finally, brain endothelial cells express ApoM, with favored secretion toward the BBB brain side, thus contributing to intra-cerebral transport of S1P. The beneficial effects of the S1P analog FTY720 in animal models of MS are coherent with a central role of ApoM in neuroinflammation control. Also, the anti-inflammatory properties of ApoA-I-containing HDLs represent an endogenous protective mechanism against MS (113). ApoA-I levels negatively correlate with disease severity, and MS patients with high levels of serum ApoA-I respond better to IFNβ therapy. The anti-inflammatory effects of this apolipoprotein are thought to be mediated, at least in part, through its prevention of LDL oxidation. ApoA-I avoids the oxLDL-mediated BBB leakage.

Regardless of demyelination being triggered by neuroinflammation, or derived from intrinsic alterations in oligodendrocytes (differentiation, myelin biogenesis, or myelin recycling), we need to keep investigating how demyelination takes place and how the myelin debris generated are dealt with. Myelin recognition and phagocytosis by resident microglia or infiltrated macrophages is a central process whose failure can trigger anomalous myelin destruction, prevent myelin clearance, or halt myelin repair. A recent gene expression analysis of MS lesions, where active demyelinating lesions are compared with inactive ones and with healthy myelinated tissue, revealed genes related to lipid binding and uptake (scavenger receptors) upregulated in the rim of chronic active lesions, where demyelination is taking place (114). Also, myelin phagocytosis control by ApoD is key for proper myelin clearance after injury (50). By regulating the presence at the injury site of lipid mediators of inflammation, and of “eat-me” signals triggering recognition of myelin debris by phagocytic cells, this Lipocalin influences the initiation of myelin phagocytosis. In addition, ApoD targeting to lysosomes and functional stabilization of lysosomal membranes (78) contributes to optimize the process of myelin degradation after autophagosome-lysosome fusion. Finally, the process of myelin reconstruction after injury is expected to require ApoD at its latest phase: myelin sheath compaction (87). It is therefore not unexpected that ApoD overexpression is detected in MS patients (115), and that its presence in MS lesions (116) is low in sclerosis plaques, particularly in inactive ones, but recovers high levels in the re-myelinating lesions when myelin reconstruction is taking place.

Myelin repair has been recently shown to depend on LRP1 (117). In addition to all signaling cascades triggered by LRP1, its action as a lipoprotein receptor (preferentially for ApoE-HDL, as mentioned above) is required for myelin repair. The cholesterol supply required for myelin sheath growth is impaired in oligodendrocytes lacking LRP1, thus hindering repair after demyelination.

Therefore, a combination of lipid-binding proteins are involved in various phases of demyelinating diseases, with effects at different levels, from BBB stability to inflammatory signaling, myelin destruction, and reconstruction. Knowledge of lipid management at each of these steps is a necessary challenge we need to take up, if we want to control myelin-related ailments.

### Lysosomal Storage Diseases and Their Inseparable Companions: Leukodystrophies

One cruel evidence that myelin–lysosome functional link is essential for human health is the existence of lysosomal storage diseases (LSDs), all of them associated to leukodystrophy or myelination problems. LSDs usually show an early onset and are suffered by children that present both systemic and nervous system-related problems and show poor survival chances. LSDs are cataloged as rare diseases, but together they add up to 1 case/8000 (118).

NPC disease has been extensively studied (119). In NPC, mutations in NPC1 and NPC2 genes cause impaired trafficking of cholesterol in neurons, after LDLR-mediated internalization, or in glial cells, where it is endogenously produced. Both proteins are located in endosomes and lysosomes and have a cholesterol-binding domain. In NPC patients, cholesterol and other lipids accumulate in late endosomes and lysosomes and are unable to travel to the plasma membrane. These lipid management alterations result in progressive neurodegeneration, starting early in cerebellar Purkinje cells (120), as well as in myelin defects, revealing the importance of cholesterol mobilization from lysosomes, both in neurons and in oligodendrocytes, to form and maintain a functional myelin sheath (121). Interestingly, concomitant with this cholesterol dyshomeostasis, genomic instability, and trisomy 21 mosaicism have been described in NPC patients (122), an effect that is thought to be a consequence of changes in membrane fluidity. Thus, lipid transport defects can also have consequences in the genetic material of cells and their correct segregation during cell division.

Niemann–Pick type A (NPA) disease is caused by a different genetic alteration, but also alters both lysosomes and myelin. It results from loss-of-function mutations in the ASM gene, leading to accumulation of sphingomyelin in lysosomes (123). NPA is an infantile disease with a rapid progression leading to premature death. It causes early neurodegeneration, particularly in vulnerable Purkinje cells, and also leads to abnormal myelin formation (124). Primarily caused by a lipid-processing enzyme deficit, this disease is also modulated by non-enzymatic lipid managing proteins, like Hsp70. As mentioned above, its lipid presenting role in lysosomes promotes ASM activity (77), and it is able to correct the lysosomal defects in cells of NPA patients (125). Analogous to Hsp70 lipid presenting role, the lysosomal Saposin B binds cerebroside sulfate and other membrane glycosphingolipids (126) to make them available to water-soluble enzymes. Defective Saposin B causes metachromatic leukodystropy, a rare LSD affecting mostly myelinating glia both in CNS and PNS, and resulting in motor and cognitive deterioration (127).

Most lysosomal dysfunctions in LSDs are primarily caused by aberrant or decreased enzymatic activities, and some, like NPC, are directly linked to non-enzymatic lipid manager proteins. It is clear that alterations in the lipid-related lysosomal functions are of particular centrality because of the many downstream consequences, including diverse forms of myelin dystrophy.

## Concluding Remarks

Lipids are essential components of all living cells requiring special management while immersed in a mostly hydrophilic environment. In addition to their energetic or signaling functions, they form structures of special relevance in the lipid-rich nervous system. Lipid-based structures are of high complexity, from apparently simple cell membranes to lipoprotein particles, intracellular lipid droplets, or complex myelin sheaths. Moreover, these structures are dynamic, serving functions as important to the nervous system as synaptic communication, nerve conduction velocity, microglial, or astroglial phagocytosis, or autophagy control of intracellular toxic products.

Lipid processing enzymes as well as lipid carriers with a variety of designs need to act in conjunction to optimize the lipid requirements of each cell type in the nervous system. In this review, we have brought the attention to non-enzymatic lipid managers and their relationship to essential processes that, when disrupted, generate a wide array of nervous system diseases. Moreover, many of the lipid-managing proteins are part of endogenous mechanisms of protection. Knowledge about all steps in each lipid-related process, the proteins involved, and the lipid species affected, should help to design potential therapies for nervous system diseases.

## Author Contributions

MG, DS, and MC-G reviewed literature and designed the manuscript. MC-G and MG wrote the manuscript. DS reviewed and edited the manuscript. The external Frontiers reviewers helped to improve the manuscript.

### Conflict of Interest

The authors declare that the research was conducted in the absence of any commercial or financial relationships that could be construed as a potential conflict of interest.

## References

[1] van Meer G Voelker DR Feigenson GW. Membrane lipids: where they are and how they behave. Nat Rev Mol Cell Biol. (2008) 9:112–24. 10.1038/nrm2330 18216768 PMC2642958

[2] Tumanov S Kamphorst JJ. Recent advances in expanding the coverage of the lipidome. Curr Opin Biotechnol. (2017) 43:127–33. 10.1016/j.copbio.2016.11.008 27915214 PMC5312421

[3] Xicoy H Wieringa B Martens GJM. The role of lipids in Parkinson's disease. Cells. (2019) 8:E27. 10.3390/cells8010027 30621069 PMC6356353

[4] Dietschy JM Turley SD. Thematic review series: brain lipids. Cholesterol metabolism in the central nervous system during early development and in the mature animal. J Lipid Res. (2004) 45:1375–97. 10.1194/jlr.R400004-JLR200 15254070

[5] Grimm MO Mett J Grimm HS Hartmann T. APP function and lipids: a bidirectional link. Front Mol Neurosci. (2017) 10:63. 10.3389/fnmol.2017.00063 28344547 PMC5344993

[6] Attwell D Mishra A Hall CN O'Farrell FM Dalkara T. What is a pericyte? J Cereb Blood Flow Metab. (2016) 36:451–5. 10.1177/0271678X15610340 26661200 PMC4759679

[7] Herndon JM Tome ME Davis TP Chapter 9—Development and maintenance of the blood–Brain barrier. In: Caplan LR Biller J Leary MC Lo EH Thomas AJ Yenari M Primer on Cerebrovascular Diseases. 2nd ed San Diego, CA: Academic Press (2017). p. 51–6.

[8] Button EB Boyce GK Wilkinson A Stukas S Hayat A Fan J. ApoA-I deficiency increases cortical amyloid deposition, cerebral amyloid angiopathy, cortical and hippocampal astrogliosis, and amyloid-associated astrocyte reactivity in APP/PS1 mice. Alzheimers Res Ther. (2019) 11:44. 10.1186/s13195-019-0497-9 31084613 PMC6515644

[9] Kajani S Curley S McGillicuddy FC. Unravelling HDL-looking beyond the cholesterol surface to the quality within. Int J Mol Sci. (2018) 19:E1971. 10.3390/ijms19071971 29986413 PMC6073561

[10] Dehouck B Fenart L Dehouck MP Pierce A Torpier G Cecchelli R. A new function for the LDL receptor: transcytosis of LDL across the blood-brain barrier. J Cell Biol. (1997) 138:877–89. 10.1083/jcb.138.4.877 9265653 PMC2138047

[11] Lane-Donovan C Philips GT Herz J. More than cholesterol transporters: lipoprotein receptors in CNS function and neurodegeneration. Neuron. (2014) 83:771–87. 10.1016/j.neuron.2014.08.005 25144875 PMC4240629

[12] Owen JB Sultana R Aluise CD Erickson MA Price TO Bu G. Oxidative modification to LDL receptor-related protein 1 in hippocampus from subjects with Alzheimer disease: implications for Abeta accumulation in AD brain. Free Radic Biol Med. (2010) 49:1798–803. 10.1016/j.freeradbiomed.2010.09.013 20869432 PMC2970765

[13] Chirackal Manavalan AP Kober A Metso J Lang I Becker T Hasslitzer K. Phospholipid transfer protein is expressed in cerebrovascular endothelial cells and involved in high density lipoprotein biogenesis and remodeling at the blood-brain barrier. J Biol Chem. (2014) 289:4683–98. 10.1074/jbc.M113.499129 24369175 PMC3931031

[14] Kober AC Manavalan APC Tam-Amersdorfer C Holmer A Saeed A Fanaee-Danesh E Implications of cerebrovascular ATP-binding cassette transporter G1 (ABCG1) and apolipoprotein M in cholesterol transport at the blood-brain barrier. Biochim Biophys Acta Mol Cell Biol Lipids. (2017) 6:573–88. 10.1016/j.bbalip.2017.03.003 28315462

[15] Weiler-Guttler H Sommerfeldt M Papandrikopoulou A Mischek U Bonitz D Frey A. Synthesis of apolipoprotein A-1 in pig brain microvascular endothelial cells. J Neurochem. (1990) 54:444–50. 10.1111/j.1471-4159.1990.tb01892.x 2105375

[16] Mockel B Zinke H Flach R Weiss B Weiler-Guttler H Gassen HG. Expression of apolipoprotein A-I in porcine brain endothelium *in vitro*. J Neurochem. (1994) 62:788–98. 10.1046/j.1471-4159.1994.62020788.x 8294940

[17] Elliott DA Weickert CS Garner B. Apolipoproteins in the brain: implications for neurological and psychiatric disorders. Clin Lipidol. (2010) 51:555–73. 10.2217/clp.10.37 21423873 PMC3058497

[18] Pincon A Thomas MH Huguet M Allouche A Colin JC Georges A. Increased susceptibility of dyslipidemic LSR+/- mice to amyloid stress is associated with changes in cortical cholesterol levels. J Alzheimers Dis. (2015) 45:195–204. 10.3233/JAD-142127 25690661

[19] Zlokovic BV Martel CL Matsubara E McComb JG Zheng G McCluskey RT. Glycoprotein 330/megalin: probable role in receptor-mediated transport of apolipoprotein J alone and in a complex with Alzheimer disease amyloid beta at the blood-brain and blood-cerebrospinal fluid barriers. Proc Natl Acad Sci USA. (1996) 93:4229–34. 10.1073/pnas.93.9.4229 8633046 PMC39517

[20] Gelissen IC Hochgrebe T Wilson MR Easterbrook-Smith SB Jessup W Dean RT. Apolipoprotein J (clusterin) induces cholesterol export from macrophage-foam cells: a potential anti-atherogenic function? Biochem J. (1998) 331:231–7. 10.1042/bj3310231 9512484 PMC1219343

[21] Zhao N Liu CC Qiao W Bu G. Apolipoprotein E, receptors, and modulation of Alzheimer's disease. Biol Psychiatry. (2018) 83:347–57. 10.1016/j.biopsych.2017.03.003 28434655 PMC5599322

[22] Pyne NJ Pyne S. Sphingosine 1-phosphate receptor 1 signaling in mammalian cells. Molecules. (2017) 22:E344. 10.3390/molecules22030344 28241498 PMC6155263

[23] Ruiz M Frej C Holmer A Guo LJ Tran S Dahlback B. High-density lipoprotein-associated apolipoprotein M limits endothelial inflammation by delivering sphingosine-1-phosphate to the sphingosine-1-phosphate receptor 1. Arterioscler Thromb Vasc Biol. (2017) 37:118–29. 10.1161/ATVBAHA.116.308435 27879252

[24] Ding F Yao J Rettberg JR Chen S Brinton RD. Early decline in glucose transport and metabolism precedes shift to ketogenic system in female aging and Alzheimer's mouse brain: implication for bioenergetic intervention. PLoS ONE. (2013) 8:e79977. 10.1371/journal.pone.0079977 24244584 PMC3823655

[25] Guzman M Blazquez C. Ketone body synthesis in the brain: possible neuroprotective effects. Prostaglandins Leukot Essent Fatty Acids. (2004) 70:287–92. 10.1016/j.plefa.2003.05.001 14769487

[26] Akerstrom B Borregaard N Flower DR Salier JP editors. Lipocalins. Georgetown, TX: Landes Bioscience (2006).

[27] Chmurzynska A. The multigene family of fatty acid-binding proteins (FABPs): function, structure and polymorphism. J Appl Genet. (2006) 47:39–48. 10.1007/BF03194597 16424607

[28] Heras-Sandoval D Pedraza-Chaverri J Perez-Rojas JM. Role of docosahexaenoic acid in the modulation of glial cells in Alzheimer's disease. J Neuroinflammation. (2016) 13:61. 10.1186/s12974-016-0525-7 26965310 PMC4787218

[29] Tachikawa M Akanuma SI Imai T Okayasu S Tomohiro T Hatanaka Y. Multiple cellular transport and binding processes of unesterified docosahexaenoic acid in outer blood-retinal barrier retinal pigment epithelial cells. Biol Pharm Bull. (2018) 41:1384–92. 10.1248/bpb.b18-00185 30175775

[30] Pan Y Short JL Choy KH Zeng AX Marriott PJ Owada Y. Fatty acid-binding protein 5 at the blood-brain barrier regulates endogenous brain docosahexaenoic acid levels and cognitive function. J Neurosci. (2016) 36:11755–67. 10.1523/JNEUROSCI.1583-16.2016 27852782 PMC6705637

[31] Puspita L Chung SY Shim JW. Oxidative stress and cellular pathologies in Parkinson's disease. Mol Brain. (2017) 10:53. 10.1186/s13041-017-0340-9 29183391 PMC5706368

[32] Pratico D. Alzheimer's disease and oxygen radicals: new insights. Biochem Pharmacol. (2002) 63:563–7. 10.1016/S0006-2952(01)00919-4 11992623

[33] Lopez-Otin C Blasco MA Partridge L Serrano M Kroemer G. The hallmarks of aging. Cell. (2013) 153:1194–217. 10.1016/j.cell.2013.05.039 23746838 PMC3836174

[34] de Magalhaes JP Curado J Church GM. Meta-analysis of age-related gene expression profiles identifies common signatures of aging. Bioinformatics. (2009) 25:875–81. 10.1093/bioinformatics/btp073 19189975 PMC2732303

[35] Dassati S Waldner A Schweigreiter R. Apolipoprotein D takes center stage in the stress response of the aging and degenerative brain. Neurobiol Aging. (2014) 35:1632–42. 10.1016/j.neurobiolaging.2014.01.148 24612673 PMC3988949

[36] Sanchez D Lopez-Arias B Torroja L Canal I Wang X Bastiani MJ. Loss of glial lazarillo, a homolog of apolipoprotein D, reduces lifespan and stress resistance in Drosophila. Curr Biol. (2006) 16:680–6. 10.1016/j.cub.2006.03.024 16581513

[37] Ganfornina MD Do Carmo S Lora JM Torres-Schumann S Vogel M Allhorn M. Apolipoprotein D is involved in the mechanisms regulating protection from oxidative stress. Aging Cell. (2008) 7:506–15. 10.1111/j.1474-9726.2008.00395.x 18419796 PMC2574913

[38] Hull-Thompson J Muffat J Sanchez D Walker DW Benzer S Ganfornina MD. Control of metabolic homeostasis by stress signaling is mediated by the lipocalin NLaz. PLoS Genet. (2009) 5:e1000460. 10.1371/journal.pgen.1000460 19390610 PMC2667264

[39] Bajo-Graneras R Ganfornina MD Martin-Tejedor E Sanchez D. Apolipoprotein D mediates autocrine protection of astrocytes and controls their reactivity level, contributing to the functional maintenance of paraquat-challenged dopaminergic systems. Glia. (2011) 59:1551–66. 10.1002/glia.21200 21688324

[40] Sanchez D Bajo-Graneras R Del Cano-Espinel M Garcia-Centeno R Garcia-Mateo N Pascua-Maestro R. Aging without Apolipoprotein D: molecular and cellular modifications in the hippocampus and cortex. Exp Gerontol. (2015) 67:19–47. 10.1016/j.exger.2015.04.003 25868396

[41] Bhatia S Knoch B Wong J Kim WS Else PL Oakley AJ. Selective reduction of hydroperoxyeicosatetraenoic acids to their hydroxy derivatives by apolipoprotein D: implications for lipid antioxidant activity and Alzheimer's disease. Biochem J. (2012) 442:713–21. 10.1042/BJ20111166 22150111

[42] Herault Y Chatelain G Brun G Michel D. V-src-induced-transcription of the avian clusterin gene. Nucleic Acids Res. (1992) 20:6377–83. 10.1093/nar/20.23.6377 1475199 PMC334530

[43] Trougakos IP So A Jansen B Gleave ME Gonos ES. Silencing expression of the clusterin/apolipoprotein j gene in human cancer cells using small interfering RNA induces spontaneous apoptosis, reduced growth ability, and cell sensitization to genotoxic and oxidative stress. Cancer Res. (2004) 64:1834–42. 10.1158/0008-5472.CAN-03-2664 14996747

[44] Franceschi C Capri M Monti D Giunta S Olivieri F Sevini F. Inflammaging and anti-inflammaging: a systemic perspective on aging and longevity emerged from studies in humans. Mech Ageing Dev. (2007) 128:92–105. 10.1016/j.mad.2006.11.016 17116321

[45] Pocivavsek A Rebeck GW. Inhibition of c-Jun N-terminal kinase increases apoE expression *in vitro* and *in vivo*. Biochem Biophys Res Commun. (2009) 387:516–20. 10.1016/j.bbrc.2009.07.048 19615334 PMC2745314

[46] Baitsch D Bock HH Engel T Telgmann R Muller-Tidow C Varga G. Apolipoprotein E induces antiinflammatory phenotype in macrophages. Arterioscler Thromb Vasc Biol. (2011) 31:1160–8. 10.1161/ATVBAHA.111.222745 21350196 PMC3529398

[47] Osburg B Peiser C Domling D Schomburg L Ko YT Voigt K. Effect of endotoxin on expression of TNF receptors and transport of TNF-alpha at the blood-brain barrier of the rat. Am J Physiol Endocrinol Metab. (2002) 283:E899–908. 10.1152/ajpendo.00436.2001 12376316

[48] Cockerill GW Rye KA Gamble JR Vadas MA Barter PJ. High-density lipoproteins inhibit cytokine-induced expression of endothelial cell adhesion molecules. Arterioscler Thromb Vasc Biol. (1995) 15:1987–94. 10.1161/01.ATV.15.11.1987 7583580

[49] Ganfornina MD Do Carmo S Martinez E Tolivia J Navarro A Rassart E. ApoD, a glia-derived apolipoprotein, is required for peripheral nerve functional integrity and a timely response to injury. Glia. (2010) 58:1320–34. 10.1002/glia.21010 20607718 PMC7165554

[50] Garcia-Mateo N Ganfornina MD Montero O Gijon MA Murphy RC Sanchez D. Schwann cell-derived Apolipoprotein D controls the dynamics of post-injury myelin recognition and degradation. Front Cell Neurosci. (2014) 8:374. 10.3389/fncel.2014.00374 25426024 PMC4227524

[51] Nikolaev A McLaughlin T O'Leary DD Tessier-Lavigne M. APP binds DR6 to trigger axon pruning and neuron death via distinct caspases. Nature. (2009) 457:981–9. 10.1038/nature07767 19225519 PMC2677572

[52] Taylor CJ Ireland DR Ballagh I Bourne K Marechal NM Turner PR. Endogenous secreted amyloid precursor protein-alpha regulates hippocampal NMDA receptor function, long-term potentiation and spatial memory. Neurobiol Dis. (2008) 31:250–60. 10.1016/j.nbd.2008.04.011 18585048

[53] Golabek A Marques MA Lalowski M Wisniewski T. Amyloid beta binding proteins *in vitro* and in normal human cerebrospinal fluid. Neurosci Lett. (1995) 191:79–82. 10.1016/0304-3940(95)11565-7 7659297

[54] Zandl-Lang M Fanaee-Danesh E Sun Y Albrecher NM Gali CC Cancar I. Regulatory effects of simvastatin and apoJ on APP processing and amyloid-beta clearance in blood-brain barrier endothelial cells. Biochim Biophys Acta Mol Cell Biol Lipids. (2018) 1863:40–60. 10.1016/j.bbalip.2017.09.008 28941799

[55] Yerbury JJ Poon S Meehan S Thompson B Kumita JR Dobson CM. The extracellular chaperone clusterin influences amyloid formation and toxicity by interacting with prefibrillar structures. FASEB J. (2007) 21:2312–22. 10.1096/fj.06-7986com 17412999

[56] Linton MF Gish R Hubl ST Butler E Esquivel C Bry WI. Phenotypes of apolipoprotein B and apolipoprotein E after liver transplantation. J Clin Invest. (1991) 88:270–81. 10.1172/JCI115288 2056122 PMC296029

[57] Huynh TV Davis AA Ulrich JD Holtzman DM. Apolipoprotein E and Alzheimer's disease: the influence of apolipoprotein E on amyloid-beta and other amyloidogenic proteins. J Lipid Res. (2017) 58:824–36. 10.1194/jlr.R075481 28246336 PMC5408619

[58] Liu Q Trotter J Zhang J Peters MM Cheng H Bao J. Neuronal LRP1 knockout in adult mice leads to impaired brain lipid metabolism and progressive, age-dependent synapse loss and neurodegeneration. J Neurosci. (2010) 30:17068–78. 10.1523/JNEUROSCI.4067-10.2010 21159977 PMC3146802

[59] Pfrieger FW Ungerer N. Cholesterol metabolism in neurons and astrocytes. Prog Lipid Res. (2011) 50:357–71. 10.1016/j.plipres.2011.06.002 21741992

[60] Huang Y Mahley RW. Apolipoprotein E: structure and function in lipid metabolism, neurobiology, and Alzheimer's diseases. Neurobiol Dis. (2014) 72:3–12. 10.1016/j.nbd.2014.08.025 25173806 PMC4253862

[61] Xu Q Bernardo A Walker D Kanegawa T Mahley RW Huang Y. Profile and regulation of apolipoprotein E (ApoE) expression in the CNS in mice with targeting of green fluorescent protein gene to the ApoE locus. J Neurosci. (2006) 26:4985–94. 10.1523/JNEUROSCI.5476-05.2006 16687490 PMC6674234

[62] Wahrle SE Jiang H Parsadanian M Legleiter J Han X Fryer JD. ABCA1 Is Required for Normal Central Nervous System ApoE Levels and for Lipidation of Astrocyte-secreted apoE. J Biol Chem. (2004) 279:40987–93. 10.1074/jbc.M407963200 15269217

[63] Koldamova R Staufenbiel M Lefterov I. Lack of ABCA1 considerably decreases brain ApoE level and increases amyloid deposition in APP23 mice. J Biol Chem. (2005) 280:43224–35. 10.1074/jbc.M504513200 16207713

[64] Hatters DM Peters-Libeu CA Weisgraber KH. Apolipoprotein E structure: insights into function. Trends Biochem Sci. (2006) 31:445–54. 10.1016/j.tibs.2006.06.008 16820298

[65] Morrow JA Hatters DM Lu B Hochtl P Oberg KA Rupp B. Apolipoprotein E4 forms a molten globule. A potential basis for its association with disease. J Biol Chem. (2002) 277:50380–5. 10.1074/jbc.M204898200 12393895

[66] Hass S Fresser F Kochl S Beyreuther K Utermann G Baier G. Physical interaction of ApoE with amyloid precursor protein independent of the amyloid Abeta region *in vitro*. J Biol Chem. (1998) 273:13892–7. 9593736

[67] Cam JA Zerbinatti CV Li Y Bu G. Rapid endocytosis of the low density lipoprotein receptor-related protein modulates cell surface distribution and processing of the beta-amyloid precursor protein. J Biol Chem. (2005) 280:15464–70. 10.1074/jbc.M500613200 15705569

[68] Huang Y-WA Zhou B Wernig M Südhof TC. ApoE2, ApoE3, and ApoE4 differentially stimulate APP transcription and Aβ secretion. Cell. (2017) 168:427–441.e421. 10.1016/j.cell.2016.12.044 28111074 PMC5310835

[69] Tokuda T Calero M Matsubara E Vidal R Kumar A Permanne B. Lipidation of apolipoprotein E influences its isoform-specific interaction with Alzheimer's amyloid beta peptides. Biochem J. (2000) 348:359–65. 10.1042/bj3480359 10816430 PMC1221074

[70] Zhu M Fink AL. Lipid binding inhibits alpha-synuclein fibril formation. J Biol Chem. (2003) 278:16873–7. 10.1074/jbc.M210136200 12621030

[71] Golovko MY Barceló-Coblijn G Castagnet PI Austin S Combs CK Murphy EJ. The role of α-synuclein in brain lipid metabolism: a downstream impact on brain inflammatory response. Mol Cell Biochem. (2009) 326:55–66. 10.1007/s11010-008-0008-y 19116775

[72] Vargas KJ Makani S Davis T Westphal CH Castillo PE Chandra SS. Synucleins regulate the kinetics of synaptic vesicle endocytosis. J Neurosci. (2014) 34:9364–76. 10.1523/JNEUROSCI.4787-13.2014 25009269 PMC4087213

[73] Ben Gedalya T Loeb V Israeli E Altschuler Y Selkoe DJ Sharon R. Alpha-synuclein and polyunsaturated fatty acids promote clathrin-mediated endocytosis and synaptic vesicle recycling. Traffic. (2009) 10:218–34. 10.1111/j.1600-0854.2008.00853.x 18980610 PMC2694501

[74] Varkey J Isas JM Mizuno N Jensen MB Bhatia VK Jao CC. Membrane curvature induction and tubulation are common features of synucleins and apolipoproteins. J Biol Chem. (2010) 285:32486–93. 10.1074/jbc.M110.139576 20693280 PMC2952250

[75] Appelqvist H Waster P Kagedal K Ollinger K. The lysosome: from waste bag to potential therapeutic target. J Mol Cell Biol. (2013) 5:214–26. 10.1093/jmcb/mjt022 23918283

[76] Sun Y Grabowski GA. Altered autophagy in the mice with a deficiency of saposin A and saposin B. Autophagy. (2013) 9:1115–6. 10.4161/auto.24919 23697974 PMC3722325

[77] Zhu H Yoshimoto T Yamashima T. Heat shock protein 70.1 (Hsp70.1) affects neuronal cell fate by regulating lysosomal acid sphingomyelinase. J Biol Chem. (2014) 289:27432–43. 10.1074/jbc.M114.560334 25074941 PMC4183783

[78] Pascua-Maestro R Diez-Hermano S Lillo C Ganfornina MD Sanchez D. Protecting cells by protecting their vulnerable lysosomes: identification of a new mechanism for preserving lysosomal functional integrity upon oxidative stress. PLoS Genet. (2017) 13:e1006603. 10.1371/journal.pgen.1006603 28182653 PMC5325589

[79] del Cano-Espinel M Acebes JR Sanchez D Ganfornina MD Lazarillo-related lipocalins confer long-term protection against type I Spinocerebellar Ataxia degeneration contributing to optimize selective autophagy. Mol Neurodegener. (2015) 10:11 10.1186/s13024-015-0009-8 25888134 PMC4374295

[80] Chan YK Sung HK Jahng JW Kim GH Han M Sweeney G. Lipocalin-2 inhibits autophagy and induces insulin resistance in H9c2 cells. Mol Cell Endocrinol. (2016) 430:68–76. 10.1016/j.mce.2016.04.006 27090568

[81] Sung HK Chan YK Han M Jahng JWS Song E Danielson E. Lipocalin-2 (NGAL) attenuates autophagy to exacerbate cardiac apoptosis induced by myocardial ischemia. J Cell Physiol. (2017) 232:2125–34. 10.1002/jcp.25672 27800610

[82] Bi F Huang C Tong J Qiu G Huang B Wu Q. Reactive astrocytes secrete lcn2 to promote neuron death. Proc Natl Acad Sci USA. (2013) 110:4069–74. 10.1073/pnas.1218497110 23431168 PMC3593910

[83] Jha MK Lee S Park DH Kook H Park KG Lee IK. Diverse functional roles of lipocalin-2 in the central nervous system. Neurosci Biobehav Rev. (2015) 49:135–56. 10.1016/j.neubiorev.2014.12.006 25511817

[84] White R Kramer-Albers EM. Axon-glia interaction and membrane traffic in myelin formation. Front Cell Neurosci. (2014) 7:284. 10.3389/fncel.2013.00284 24431989 PMC3880936

[85] Fields RD. A new mechanism of nervous system plasticity: activity-dependent myelination. Nat Rev Neurosci. (2015) 16:756–67. 10.1038/nrn4023 26585800 PMC6310485

[86] Stadelmann C Timmler S Barrantes-Freer A Simons M. Myelin in the central nervous system: structure, function, and pathology. Physiol Rev. (2019) 99:1381–431. 10.1152/physrev.00031.2018 31066630

[87] Garcia-Mateo N Pascua-Maestro R Perez-Castellanos A Lillo C Sanchez D Ganfornina MD. Myelin extracellular leaflet compaction requires apolipoprotein D membrane management to optimize lysosomal-dependent recycling and glycocalyx removal. Glia. (2018) 66:670–87. 10.1002/glia.23274 29222871

[88] Klosinski LP Yao J Yin F Fonteh AN Harrington MG Christensen TA. White matter lipids as a ketogenic fuel supply in aging female brain: implications for Alzheimer's disease. EBioMedicine. (2015) 2:1888–904. 10.1016/j.ebiom.2015.11.002 26844268 PMC4703712

[89] Chun BY Kim JH Nam Y Huh MI Han S Suk K. Pathological involvement of astrocyte-derived lipocalin-2 in the demyelinating optic neuritis. Invest Ophthalmol Vis Sci. (2015) 56:3691–8. 10.1167/iovs.15-16851 26047170

[90] Ranganathan S Noyes NC Migliorini M Winkles JA Battey FD Hyman BT. LRAD3, a novel low-density lipoprotein receptor family member that modulates amyloid precursor protein trafficking. J Neurosci. (2011) 31:10836–46. 10.1523/JNEUROSCI.5065-10.2011 21795536 PMC3189500

[91] Cam JA Zerbinatti CV Knisely JM Hecimovic S Li Y Bu G. The low density lipoprotein receptor-related protein 1B retains beta-amyloid precursor protein at the cell surface and reduces amyloid-beta peptide production. J Biol Chem. (2004) 279:29639–46. 10.1074/jbc.M313893200 15126508

[92] Kanekiyo T Cirrito JR Liu CC Shinohara M Li J Schuler DR. Neuronal clearance of amyloid-beta by endocytic receptor LRP1. J Neurosci. (2013) 33:19276–83. 10.1523/JNEUROSCI.3487-13.2013 24305823 PMC3850043

[93] Bell RD Sagare AP Friedman AE Bedi GS Holtzman DM Deane R. Transport pathways for clearance of human Alzheimer's amyloid beta-peptide and apolipoproteins E and J in the mouse central nervous system. J Cereb Blood Flow Metab. (2007) 27:909–18. 10.1038/sj.jcbfm.9600419 17077814 PMC2853021

[94] Foster EM Dangla-Valls A Lovestone S Ribe EM Buckley NJ. Clusterin in Alzheimer's disease: mechanisms, genetics, and lessons from other pathologies. Front Neurosci. (2019) 13:164. 10.3389/fnins.2019.00164 30872998 PMC6403191

[95] Sweeney MD Sagare AP Zlokovic BV. Blood-brain barrier breakdown in Alzheimer disease and other neurodegenerative disorders. Nat Rev Neurol. (2018) 14:133–50. 10.1038/nrneurol.2017.188 29377008 PMC5829048

[96] Khoonsari PE Haggmark A Lonnberg M Mikus M Kilander L Lannfelt L. Analysis of the cerebrospinal fluid proteome in Alzheimer's disease. PLoS ONE. (2016) 11:e0150672. 10.1371/journal.pone.0150672 26950848 PMC4780771

[97] Keeney JT Swomley AM Forster S Harris JL Sultana R Butterfield DA. Apolipoprotein A-I: insights from redox proteomics for its role in neurodegeneration. Proteomics Clin Appl. (2013) 7:109–22. 10.1002/prca.201200087 23027708 PMC3760000

[98] Montagne A Zhao Z Zlokovic BV. Alzheimer's disease: a matter of blood–brain barrier dysfunction? J Exp Med. (2017) 214:3151. 10.1084/jem.20171406 29061693 PMC5679168

[99] Winkler EA Nishida Y Sagare AP Rege SV Bell RD Perlmutter D. GLUT1 reductions exacerbate Alzheimer's disease vasculo-neuronal dysfunction and degeneration. Nat Neurosci. (2015) 18:521–30. 10.1038/nn.3966 25730668 PMC4734893

[100] Emamzadeh FN. Role of apolipoproteins and alpha-synuclein in Parkinson's disease. J Mol Neurosci. (2017) 62:344–55. 10.1007/s12031-017-0942-9 28695482 PMC5541107

[101] Refolo V Stefanova N. Neuroinflammation and glial phenotypic changes in alpha-synucleinopathies. Front Cell Neurosci. (2019) 13:263. 10.3389/fncel.2019.00263 31263402 PMC6585624

[102] Bartels T Choi JG Selkoe DJ. alpha-Synuclein occurs physiologically as a helically folded tetramer that resists aggregation. Nature. (2011) 477:107–10. 10.1038/nature10324 21841800 PMC3166366

[103] Fauvet B Mbefo MK Fares MB Desobry C Michael S Ardah MT. alpha-Synuclein in central nervous system and from erythrocytes, mammalian cells, and *Escherichia coli* exists predominantly as disordered monomer. J Biol Chem. (2012) 287:15345–64. 10.1074/jbc.M111.318949 22315227 PMC3346117

[104] Shamoto-Nagai M Hisaka S Naoi M Maruyama W. Modification of alpha-synuclein by lipid peroxidation products derived from polyunsaturated fatty acids promotes toxic oligomerization: its relevance to Parkinson disease. J Clin Biochem Nutr. (2018) 62:207–12. 10.3164/jcbn.18-25 29892158 PMC5990400

[105] Carreras I Garrett-Young R Ullman MD Eisenhauer PB Fine RE Wells JM. Upregulation of clusterin/apolipoprotein J in lactacystin-treated SH-SY5Y cells. J Neurosci Res. (2005) 79:495–502. 10.1002/jnr.20374 15635600

[106] Sasaki K Doh-ura K Wakisaka Y Iwaki T. Clusterin/apolipoprotein J is associated with cortical Lewy bodies: immunohistochemical study in cases with alpha-synucleinopathies. Acta Neuropathol. (2002) 104:225–30. 10.1007/s00401-002-0546-4 12172907

[107] Ordonez C Navarro A Perez C Astudillo A Martinez E Tolivia J. Apolipoprotein D expression in substantia nigra of Parkinson disease. Histol Histopathol. (2006) 21:361–6. 10.14670/HH-21.361 16437381

[108] Navarro A Mendez E Diaz C del Valle E Martinez-Pinilla E Ordonez C. Lifelong expression of apolipoprotein D in the human brainstem: correlation with reduced age-related neurodegeneration. PLoS ONE. (2013) 8:e77852. 10.1371/journal.pone.0077852 24167586 PMC3805570

[109] Pascua-Maestro R Gonzalez E Lillo C Ganfornina MD Falcon-Perez JM Sanchez D. Extracellular vesicles secreted by astroglial cells transport apolipoprotein D to neurons and mediate neuronal survival upon oxidative stress. Front Cell Neurosci. (2019) 12:526. 10.3389/fncel.2018.00526 30687015 PMC6335244

[110] Emamzadeh FN Aojula H McHugh PC Allsop D. Effects of different isoforms of apoE on aggregation of the alpha-synuclein protein implicated in Parkinson's disease. Neurosci Lett. (2016) 618:146–51. 10.1016/j.neulet.2016.02.042 26921451

[111] Blaho VA Galvani S Engelbrecht E Liu C Swendeman SL Kono M. HDL-bound sphingosine-1-phosphate restrains lymphopoiesis and neuroinflammation. Nature. (2015) 523:342–6. 10.1038/nature14462 26053123 PMC4506268

[112] Hajny S Christoffersen C. A novel perspective on the ApoM-S1P axis, highlighting the metabolism of ApoM and its role in liver fibrosis and neuroinflammation. Int J Mol Sci. (2017) 18:E1636. 10.3390/ijms18081636 28749426 PMC5578026

[113] Gardner LA Levin MC. Importance of apolipoprotein A-I in multiple sclerosis. Front Pharmacol. (2015) 6:278. 10.3389/fphar.2015.00278 26635608 PMC4654019

[114] Hendrickx DAE van Scheppingen J van der Poel M Bossers K Schuurman KG van Eden CG. Gene expression profiling of multiple sclerosis pathology identifies early patterns of demyelination surrounding chronic active lesions. Front Immunol. (2017) 8:1810. 10.3389/fimmu.2017.01810 29312322 PMC5742619

[115] Reindl M Knipping G Wicher I Dilitz E Egg R Deisenhammer F. Increased intrathecal production of apolipoprotein D in multiple sclerosis. J Neuroimmunol. (2001) 119:327–32. 10.1016/S0165-5728(01)00378-2 11585636

[116] Navarro A Rioseras B Del Valle E Martinez-Pinilla E Astudillo A Tolivia J. Expression pattern of myelin-related apolipoprotein D in human multiple sclerosis lesions. Front Aging Neurosci. (2018) 10:254. 10.3389/fnagi.2018.00254 30186153 PMC6110904

[117] Lin JP Mironova YA Shrager P Giger RJ. LRP1 regulates peroxisome biogenesis and cholesterol homeostasis in oligodendrocytes and is required for proper CNS myelin development and repair. Elife. (2017) 6:e30498. 10.7554/eLife.30498 29251594 PMC5752207

[118] Renaud DL. Lysosomal disorders associated with leukoencephalopathy. Semin Neurol. (2012) 32:51–4. 10.1055/s-0032-1306386 22422206

[119] Pfeffer SR. NPC intracellular cholesterol transporter 1 (NPC1)-mediated cholesterol export from lysosomes. J Biol Chem. (2019) 294:1706–9. 10.1074/jbc.TM118.004165 30710017 PMC6364775

[120] Tang Y Li H Liu JP. Niemann-pick disease type C: from molecule to clinic. Clin Exp Pharmacol Physiol. (2010) 37:132–40. 10.1111/j.1440-1681.2009.05235.x 19566836

[121] Yu T Lieberman AP. Npc1 acting in neurons and glia is essential for the formation and maintenance of CNS myelin. PLoS Genet. (2013) 9:e1003462. 10.1371/journal.pgen.1003462 23593041 PMC3623760

[122] Granic A Potter H. Mitotic spindle defects and chromosome mis-segregation induced by LDL/cholesterol-implications for Niemann-Pick C1, Alzheimer's disease, and atherosclerosis. PLoS ONE. (2013) 8:e60718. 10.1371/journal.pone.0060718 23593294 PMC3625184

[123] Gabande-Rodriguez E Boya P Labrador V Dotti CG Ledesma MD. High sphingomyelin levels induce lysosomal damage and autophagy dysfunction in Niemann Pick disease type A. Cell Death Differ. (2014) 21:864–75. 10.1038/cdd.2014.4 24488099 PMC4013520

[124] Ledesma MD Prinetti A Sonnino S Schuchman EH. Brain pathology in Niemann Pick disease type A: insights from the acid sphingomyelinase knockout mice. J Neurochem. (2011) 116:779–88. 10.1111/j.1471-4159.2010.07034.x 21214563 PMC3059095

[125] Kirkegaard T Roth AG Petersen NH Mahalka AK Olsen OD Moilanen I. Hsp70 stabilizes lysosomes and reverts Niemann-Pick disease-associated lysosomal pathology. Nature. (2010) 463:549–53. 10.1038/nature08710 20111001

[126] Fluharty CB Johnson J Whitelegge J Faull KF Fluharty AL. Comparative lipid binding study on the cerebroside sulfate activator (saposin B). J Neurosci Res. (2001) 63:82–9. 10.1002/1097-4547(20010101)63:1<82::AID-JNR10>3.0.CO;2-D 11169617

[127] van Rappard DF Boelens JJ Wolf NI. Metachromatic leukodystrophy: disease spectrum and approaches for treatment. Best Pract Res Clin Endocrinol Metab. (2015) 29:261–73. 10.1016/j.beem.2014.10.001 25987178

